# The ^40^Ca^+^ ion optical clock

**DOI:** 10.1093/nsr/nwaa119

**Published:** 2020-06-05

**Authors:** Kelin Gao

**Affiliations:** Wuhan Institute of Physics and Mathematics, Chinese Academy of Sciences, China

Ever since the mid-twentieth century, atomic clocks have become the most accurate time and frequency standards known. In 1967, the 13th CGPM (General Conference of Weights and Measures) decided to replace the definition of the second by ‘the duration of 9 192 631 770 periods of the radiation corresponding to the transition between two hyperfine levels of the ground state of the Cs-133 atom’. Optical atomic clocks are the next generation of atomic clocks with clock frequencies >10 000 times higher (100–1000 THz) and lie in the optical spectral range. Their accuracy could be 4 orders of magnitude better. Nowadays, the best optical atomic clocks have surpassed the best Cs fountain clocks by two orders of magnitude in both accuracy and stability (Fig. 1) [1–4]. Scientists believe the definition of the second will be referenced to the optical atomic clock transition frequencies in the near future. There are different kinds of optical clocks, including the neutral atomic lattice clocks referenced to Sr, Yb or Hg atoms and single-ion clocks referenced to Sr^+^, Hg^+^, Yb^+^, Al^+^, Ca^+^, etc. For different referenced atoms or ions, different technologies have been used, so each one of them has its own advantages and disadvantages [1]. Which type of optical clock will be the best? No one really knows. However, the race to find the best clock of the future has already started.

**Figure 1.**
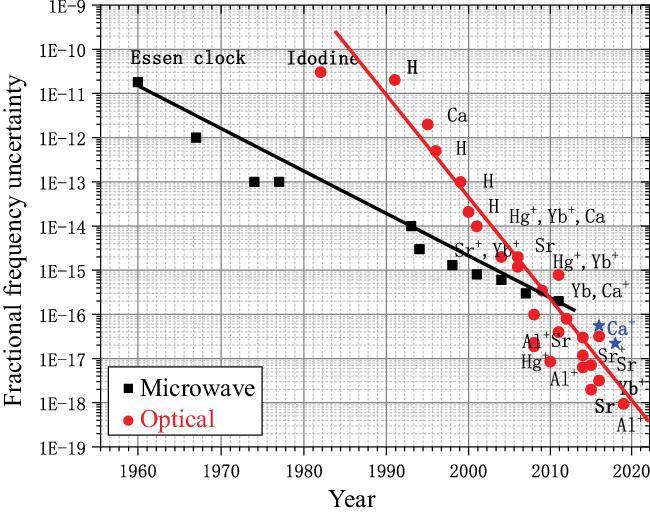
Uncertainties of microwave clocks and optical clocks. The fast development of optical clocks is shown, especially in the last two decades. The best optical atomic clocks have surpassed the best Cs fountain clocks by 2 orders of magnitude nowadays. Black squares represent the microwave clocks, red circles represent the optical clocks and blue stars represent the Ca^+^ clock built in Wuhan Institute of Physics and Mathematics, Chinese Academy of Sciences (WIPM).

Single ions can be produced in a well-controlled environment, with its frequency reproducible anywhere on Earth or even in space. Back in 1982, H. Dehmelt proposed that single ions might be used to build a clock whose transition frequency can be measured with a fractional uncertainty of 10^−18^. Since then, scientists have been working on building such high-precision clocks. Nowadays, the PTB Yb^+^ optical clock has achieved a systematic uncertainty of 3.0 × 10^−18^ [5] and, recently, the systematic uncertainty of the Al^+^ optical clock of NIST has reached a level of 9.4 × 10^−19^ [6], which is the most accurate atomic optical clock so far. In China, different kinds of optical clocks are also under development, with ultracold neutral atoms or single ions such as Sr, Yb, Ca, Hg, Al^+^, Ca^+^, Hg^+^, In^+^ and Ba^+^.

Among the different kinds of ion candidates, Ca^+^ has its advantages: a simple laser system—all the lasers can be obtained by choosing diode lasers, optical frequency doubling not needed, quadrupole shift eliminated with a simple technique, existence of magic trapping the rf frequency for greatly suppressing the micromotion shifts, stably trapped at room temperature, etc. The research of optical clocks and quantum information based on Ca^+^ is being carried out all over the world. In recent years, the measurements of the Ca^+^ clock transition frequency have been made by Innsbruck University in Austria, NICT of Japan and Wuhan Institute of Physics and Mathematics, Chinese Academy of Sciences (WIPM).

The Ca^+^-clock research in WIPM has been going on for more than two decades. Single Ca^+^ is trapped in the Paul traps and then laser cooled, successfully locked to the 729-nm probe laser to an ultra-stable cavity with a fineness of >200 000 afterwards. The probe laser has been locked to the ion transitions. There are a variety of potential sources of systematic shift that might be associated with the Ca^+^ clock; the systematic effects should be considered generally to include the Doppler and Stark shifts caused by both the micromotion and the secular motion, the blackbody radiation Stark shift, the Zeeman shift, ac Stark shift (light shift), quadrupole shift, etc. The linear Zeeman shift, the quadruple shift and the tensor part of the Stark shift would be canceled out by averaging six spectral-line frequencies.

**Table 1.** The Ca^+^-clock systematic-uncertainty budget table (unit in 10^−18^).

	Fractional	Fractional
Contribution	frequency shift	frequency uncertainty
BBR field evaluation (temperature)	863	19
BBR coefficient (Δ*α*_0_)	0	0.3
Excess micromotion	0	0.4
Second-order Doppler (thermal)	−5.0	2.5
ac Stark shift	1.2	1.3
Residual quadrupole	0	2.3
Zeeman effect	0	1.5
Servo	0.0	3.0
Total	859	20

Like most ion-based clocks, the micromotion shifts are one of the dominant systematic-uncertainty sources, which can be as large as 10^−14^∼10^−13^ if not carefully compensated. At WIPM, the micromotion shifts were minimized and evaluated using the rf-photon correlation technique and the observation of the ion imaged on a CCD camera [7]. Recently, new vacuum chambers have been built and one can probe the micromotion sidebands in three dimensions; this method is much more accurate. By keeping the ion trapped at the ‘magic’ rf trapping field at ∼24.8 MHz, the micromotion-shifts uncertainty can be easily suppressed to <1 × 10^−18^ [8].

Another important systematic shift is called the blackbody radiation shift. This shift is well known and can be precisely calculated if one knows the exact environmental temperature sensed by the ion and the related atomic constant: the static differential polarizability. This constant is difficult to calculate with very high precision, but it can be indirectly measured by measuring the ‘magic’ rf trapping frequency. The ‘magic’ rf trapping frequency is precisely measured with ∼2 orders of magnitude better precision than the calculations; the blackbody radiation shift due to atomic constant uncertainty is then reduced to <1 × 10^−18^ for a room-temperature-based Ca^+^ clock [8].

With further improvements made to reduce the servo error, the uncertainty of the evaluation of the systematic shifts has achieved a level of 2.0 × 10^−17^ (Table 1), currently limited by the blackbody radiation field evaluation uncertainty of 1.6 K.

Besides uncertainty, stability is also important for clocks. In recent years, WIPM has introduced the state preparation technique—a faster and more efficient control system is implemented; Ramsey interrogation is also introduced, making the clock stability approximately four times better than in the year 2016 [7]. A frequency comparison between two clocks shows that the stability of each single clock has reached 7 × 10^−18^ in ∼500 000 s of averaging time (Fig. 2).

**Figure 2.**
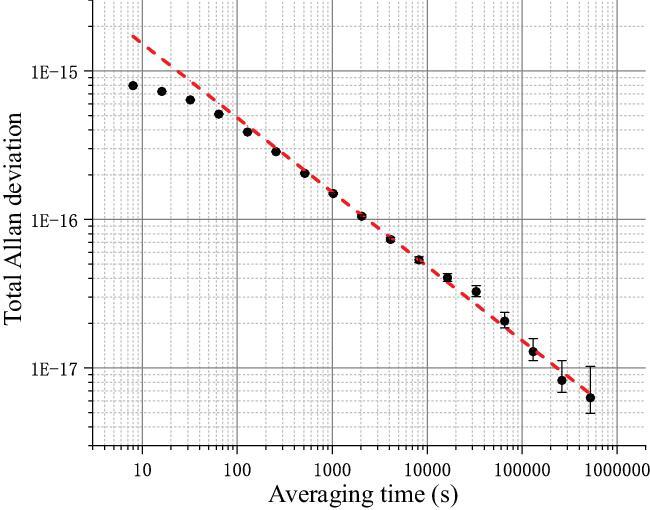
The total Allan deviation measured for a single clock (3 Feburary–6 March 2019). The result of comparison between the two clocks is divided by $\sqrt 2 $ to represent the stability for a single clock. The red dashed line shows the $1/\sqrt \tau $ fit of the data. The stability for a single clock is measured as $4.9(1) \times {10^{ - 15}}/\sqrt \tau $ and reaches $6.3({{+ 3.9}\atop{ - 1.4}}) \times {10^{ - 18}}$ in an averaging time of 524 000 s.

Ca^+^ is also a candidate for secondary representation of the SI second. In the years 2012, 2015 and 2017, the measurement results of WIPM, NICT and Innsbruck University were adopted by CIPM and the recommended frequency of the Ca^+^-clock transition has been revised three times. The uncertainty of the recommended value is reduced from 4 × 10^−14^ to 2.4 × 10^−15^ [Consultative Committee for Time and Frequency (CCTF) Reports-19 (2012), 20 (2015) and 21 (2017)].

In the future, further study on the evaluation of the BBR field is required and we are now working on housing the ion trap in a liquid-nitrogen-temperature environment to obtain further reductions in the BBR-shift uncertainty. With reductions in the shifts and their uncertainties mentioned above, a ^40^Ca^+^-ion clock with an uncertainty at the 10^−18^ level can be achieved. Furthermore, the stability is still ∼10 times worse than the quantum projection noise (QPN) limit of the single Ca^+^ clock, mainly limited by our clock-laser stability of 8 × 10^−16^. Clock lasers with a stability of <3 × 10^−16^ at 1∼300 s are needed to reach the QPN limit. To further improve the ion-clock stability, one has to build a clock referenced to multiple ions. Optical trapping of the ions under the magic wavelength would be one of the options [9]. Like the PTB portable Sr clock [10], one of the WIPM Ca^+^ clocks has been moved to a container that can be easily moved by a truck. A comparison experiment is in progress. Thanks to their simplicity, it might be easier to make portable Ca^+^ clocks—even space ones. Hopefully, in the near future, highly accurate and portable Ca^+^ clocks will find applications in mapping Earth's gravitational potential, testing fundamental physics and navigation.

## Supplementary Material

nwaa119_Supplemental_Files
