# Supplementary material for: The 40Ca+ ion optical clock
Source: Natl Sci Rev. 2020 Jun 5;7(12):1799–801. doi: 10.1093/nsr/nwaa119 (PMC8290938; doi:10.1093/nsr/nwaa119)
Supplement: nwaa119_Supplemental_Files [file nwaa119_supplemental_files.zip › supplementary_data/SM-Efficient network immunization under limited knowledge.pdf]

Supplementary Materials - Efficient network immunization under limited knowledge

Yangyang Liu,[1,*] Hillel Sanhedrai,[2,*] GaoGao Dong,[3,†] Louis M. Shekhtman,[2,4,†] Fan Wang,[2] Sergey V. Buldyrev,[5] and Shlomo Havlin[2,†]

[1]*Department of Systems Science, National University of Defense Technology, Changsha, Hunan 410073, China*
[2]*Department of Physics, Bar-Ilan University, Ramat Gan 5290002, Israel*
[3]*Faculty of Science, Jiangsu University, Zhenjiang, Jiangsu 212013, China*
[4]*Networks Science Institute, Northeastern University, Boston, MA 02115*
[5]*Department of Physics, Yeshiva University, New York, New York 10033, USA*
(Dated: September 1, 2020)

I. NOTATIONS

Unless particularly specified, the notations used in this paper are illustrated in Table I. And symbols in the derivation are also described in detail on where they first appear in the manuscript.

TABLE I. Notations used in this paper.

**Notations**	**Descriptions**
$\vert \cdot \vert$	The cardinality of a set.
G	A network.
V	The set of nodes in a network.
E	The set of edges in a network.
N	The number of nodes in a network.
$P(k,t)$	The degree distribution of a network at time t.
$F(k,t)$	The cumulative degree distribution of a network at time t.
p	The fraction of remaining nodes in a network.
P_∞	The fraction of giant component in a network.
p_c	The critical threshold where the giant component first occurs.

II. DERIVATION DETAILS

A. Derivation of $P_r(k,t)$

In Eq. (1), $F(k,t)$ is the probability that the degree of a randomly chosen node is less or equal to k at time t. According to our efficient immunization strategy, we randomly select n nodes and choose the node with highest degree to immunize. Then a node with degree k is immunized at time t under condition that it is chosen at least once and the degree of rest chosen nodes is less than k. Hence the probability $P_r(k,t)$ is

$$\begin{aligned} P_r(k,t) &= \sum_{m=1}^{n}[\frac{n!}{m!(n-m)!}P(k,t)^m(\sum_{s=0}^{k-1}P(s,t))^{n-m}] \\ &= (\sum_{s=0}^{k}P(s,t))^n - (\sum_{s=0}^{k-1}P(s,t))^n \\ &= F(k,t)^n - F(k-1,t)^n. \end{aligned} \tag{S1}$$

Another easy way to show this is that the maximal degree is less or equal to k among n nodes if and only if all degrees are lower or equal to k. Thus, $F_r(k,t) = F(k,t)^n$. Then, the probability $P_r(k,t)$ can be obtained from its cumulative probability $F_r(k,t)$,

$$P_r(k,t) = F(k,t)^n - F(k-1,t)^n. \tag{S2}$$

B. Solution of the ODE for $F(k,t)$

Consider the ODE

$$-F + (N-t)\dot{F} = -F^n, \tag{S3}$$

where $F = F(k,t)$, with the initial condition

$$F(k, t=0) = F(k). \tag{S4}$$

Using separation of variables we get

$$\frac{\mathrm{d}F}{F-F^n} = \frac{\mathrm{d}t}{N-t}.$$

Thus,

$$\begin{aligned} \frac{1}{n-1}\log\left|\frac{F^n}{F^n-F}\right| &= -\log(N-t) + C_1 \\ \left|\frac{F^n}{F^n-F}\right| &= e^{(n-1)(-\log(N-t)+C_1)} \\ F^{1-n} &= 1 + C_2 e^{(n-1)\log(N-t)} \\ F &= \left(1 + C_2 e^{(n-1)\log(N-t)}\right)^{-\frac{1}{n-1}} \end{aligned}$$

---

* Y. Liu and H. Sanhedrai contributed equally to this work
† To whom correspondence may be addressed. Email: dfocus.gao@gmail.com, lsheks@gmail.com, havlins@gmail.com

Recalling that $F(k,t=0)=F(k)$, and substituting $t=0$ gives

$$F(k)^{1-n} = 1 + C_2 e^{(n-1)\log(N)}$$
$$C_2 = \left(F(k)^{1-n}-1\right)e^{-(n-1)\log(N)}.$$

As a result,

$$F(k,t) = \left(1+\left(F(k)^{1-n}-1\right)e^{(n-1)\log[(N-t)/N]}\right)^{-\frac{1}{n-1}}, \tag{S5}$$

or

$$F_p(k) = \left(1+\left(F(k)^{1-n}-1\right)p^{n-1}\right)^{-\frac{1}{n-1}}, \tag{S6}$$

where $F_p(k)$ is the cumulative distribution of degree of the occupied nodes after removing $1-p$ fraction of nodes. For $n=1$, the solution of the ODE is $F=Const.$, namely $F_p(k)=F(k)$ as expected. Also Eq. (S6) converges to it if we take the limit $n\to 1$.

C. Derivation of the exponential convergence of p_c for large n

First, we find $F_p(k)$ and $P_p(k)$ for large n. Examining Eq. (S6) where $n\to\infty$, as elaborated below, we recognize two behaviors depending on $F(k)<p$ or $F(k)>p$. For the leading term

$$F_p^{\infty}(k) = \begin{cases} \frac{F(k)}{p}, & F(k)<p \\ 1, & F(k)>p \end{cases} \tag{S7}$$

See Fig. S1 for illustration.

Let us denote the next term as

$$F_p(k) = F_p^{\infty}(k) + \epsilon(k,p,n) \tag{S8}$$

Then, we approach finding the next term, ϵ, in each range of degree k.

For small k, such that $F(k)<p$,

$$\begin{aligned} F_p(k) &\approx \left(1+\left(\frac{p}{F}\right)^n - p^n\right)^{-\frac{1}{n}} \approx \left(1+\left(\frac{p}{F}\right)^n\right)^{-\frac{1}{n}} \\ &= \frac{F}{p}\left(1+\left(\frac{F}{p}\right)^n\right)^{-\frac{1}{n}} \\ &= \frac{F}{p}\exp\left[-\frac{1}{n}\log\left(1+\left(\frac{F}{p}\right)^n\right)\right] \\ &\approx \frac{F}{p}\exp\left[-\frac{1}{n}\left(\frac{F}{p}\right)^n\right] \approx \frac{F(k)}{p}\left[1-\frac{1}{n}\left(\frac{F(k)}{p}\right)^n\right] \\ &\approx \frac{F(k)}{p} - \frac{1}{n}e^{-(n+1)[\log p - \log F(k)]} \end{aligned} \tag{S9}$$

For the particular case that there exists a degree satisfying $\boldsymbol{F(k)=p}$,

$$\begin{aligned} F_p(k) &\approx \left(1+\left(\frac{p}{F}\right)^n - p^n\right)^{-\frac{1}{n}} \\ &\approx 2^{-\frac{1}{n}} = e^{-\frac{1}{n}\log 2} \approx 1-\frac{\log 2}{n} \end{aligned} \tag{S10}$$

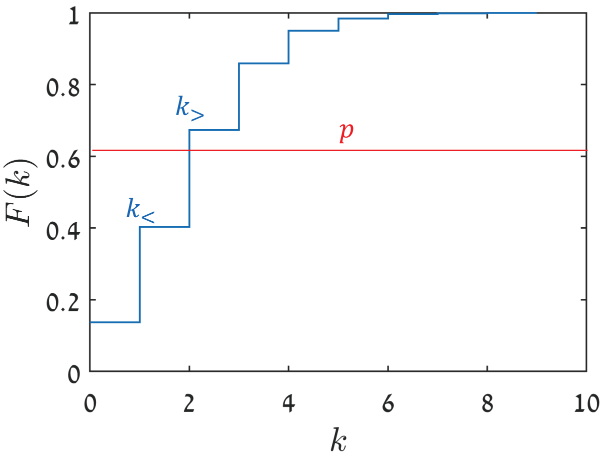

FIG. S1. **Illustration of theory for $F_p(k)$ for large n.** For small k, $F(k)<p$, $F_p(k)\to F(k)/p$, and for large k, $F(k)>p$, $F_p(k)\to 1$. The dashed horizontal line is at the value of p. $k_<$ and $k_>$ are the consecutive degrees for which $F(k_<)<p<F(k_>)$ respectively. For determining the decay rate α in Eq. (S19), one should take $p=p_c$, and find the minimum of $|\log(F(k)/p_c^{\infty})|$ among all k, which is in fact among $k_<$ and $k_>$.

For the limit $\boldsymbol{F(k)\to p}$ such that $(p/F(k))^n\to 1$, let us define $\zeta = 1-p/F$ which is small. Then,

$$\begin{aligned} (p/F)^n &= (1-\zeta)^n = \exp(n\ln(1-\zeta)) \\ &\approx 1+n\ln(1-\zeta) \approx 1-\zeta n \\ &= 1-n(1-p/F). \end{aligned}$$

Thus,

$$\begin{aligned} F_p(k) &\approx \left(1+\left(\frac{p}{F}\right)^n - p^n\right)^{-\frac{1}{n}} \\ &\approx \left(2-n(1-p/F)-p^n\right)^{-\frac{1}{n}} \\ &\approx \exp\left[-\frac{1}{n}\ln\left(2-n(1-p/F)-p^n\right)\right] \\ &\approx \exp\left[-\frac{1}{n}\ln 2 - \frac{1}{n}\ln\left(1-n(1-p/F)/2-p^n/2\right)\right] \\ &\approx \exp\left[-\frac{\ln 2}{n} + \frac{1-p/F}{2} + \frac{p^n}{2n}\right] \\ &\approx 1-\frac{\ln 2}{n} + \frac{1-p/F}{2} + \frac{p^n}{2n} \end{aligned} \tag{S11}$$

For large k, namely $p < F(k) < 1$,

$$\begin{aligned} F_p(k) &\approx \left(1+\left(\frac{p}{F}\right)^n - p^n\right)^{-\frac{1}{n}} \\ &= \exp\left[-\frac{1}{n}\log\left(1+\left(\frac{p}{F}\right)^n - p^n\right)\right] \\ &\approx \exp\left[-\frac{1}{n}\left(\frac{p}{F}\right)^n + \frac{p^n}{n}\right] \\ &\approx 1-\frac{1}{n}\left(\frac{p}{F(k)}\right)^n + \frac{p^n}{n} \\ &\approx 1-\frac{1}{n}\exp\left(-n\left[\log F(k) - \log p\right]\right). \end{aligned} \tag{S12}$$

In the limit $F(k) \to 1$, we should keep the last term above, p^n/n, in order to get $F_p(k) \to 1$. In more detail, if $F(k)^n \to 1$ we can denote a small term $\xi(k) = 1 - F(k)$, which satisfies

$$\begin{aligned} F(k)^{-n} &= \exp(-n\ln F(k)) \approx 1 - n\ln F(k) \\ &= 1 - n\ln(1-\xi(k)) \approx 1 + n\xi(k). \end{aligned}$$

Substituting this into Eq. (S12) gives

$$\begin{aligned} F_p(k) &\approx 1-\frac{1}{n}\left(\frac{p}{F(k)}\right)^n + \frac{p^n}{n} \\ &\approx 1-\xi(k)p^n = 1 - p^n(1-F(k)). \end{aligned} \tag{S13}$$

Finally, for $F(k) = 1$, it easy to see that also $F_p(k) = 1$.

To summarize, the next term of $F_p(k)$ is

$$\epsilon(k,p,n) = \begin{cases} -\frac{1}{n}e^{-\alpha_k n}, & F(k) < 1 \\ 0, & F(k) = 1 \end{cases}, \tag{S14}$$

where $\alpha_k = |\log\left[p/F(k)\right]|$.
If there are degrees which obey the limits $F(k)^n \to 1$, or $[F(k)/p]^n \to 1$, then they satisfy

$$\epsilon(k,p,n) = \begin{cases} -\frac{1}{n}\left(\ln 2 - n\left[1-\frac{p}{F(k)}\right]\right), & \left(\frac{p}{F(k)}\right)^n \to 1 \\ -p^n(1-F(k)), & F(k)^n \to 1 \end{cases}. \tag{S15}$$

Having an approximation for $F_p(k)$, we approach now to find p_c using the equation for criticality,

$$1 = \frac{p}{\langle k\rangle}\sum_{k=0}^{\infty} k(k-1)P_p(k). \tag{S16}$$

Before, we denote several special degrees: the degrees where $F(k)$ is close to p, and the maximal degree, as following

$$\begin{aligned} k_< &= \max_{F(k)<p} k, \\ k_> &= \min_{F(k)\geq p} k, \\ K &= \min_{F(k)=1} k. \end{aligned}$$

Plugging Eqs. (S7),(S14),(S8) into Eq. (S16), and using change of summation indexes, one can obtain

$$\begin{aligned} \frac{\langle k\rangle}{p_c} &= \sum_{k=2}^{\infty} k(k-1)\Delta F_p(k) \\ &= \sum_{k=2}^{\infty} k(k-1)\Delta F_p^{\infty}(k) + \sum_{k=2}^{K} k(k-1)\Delta\epsilon(k,p_c,n) \\ &= \sum_{k=2}^{k_<} k(k-1)\frac{P(k)}{p_c} + \sum_{k=k_>+1}^{\infty} k(k-1)\cdot 0 \\ &+ k_>k_<\left[1-\frac{F(k_<)}{p_c}\right] - 2\sum_{k=1}^{K} k\epsilon(k,p_c,n). \end{aligned}$$

Hence,

$$\begin{aligned} \frac{\langle k\rangle}{p_c} &= \sum_{k=2}^{k_<} k(k-1)\frac{P(k)}{p_c} \\ &+ k_>k_<\left[1-\frac{F(k_<)}{p_c}\right] - 2\sum_{k=1}^{K} k\epsilon(k,p_c,n) \end{aligned} \tag{S17}$$

and for $n \to \infty$, for the leading term, p_c^{∞},

$$\begin{aligned} \frac{\langle k\rangle}{p_c^{\infty}} &= \sum_{k=2}^{k_<} k(k-1)\frac{P(k)}{p_c^{\infty}} + k_>k_<\left[1-\frac{F(k_<)}{p_c^{\infty}}\right] \\ p_c^{\infty} &= F(k_<) + \frac{1}{k_>k_<}\left(\langle k\rangle - \sum_{k=2}^{k_<} k(k-1)P(k)\right), \end{aligned}$$

where $k_<$ and $k_>$ are determined by p_c^{∞}, but are independent of n.
For the next term, returning to the equation (S17) and obtain

$$p_c = p_c^{\infty} + \frac{2p_c^{\infty}}{k_>k_<}\sum_{k=1}^{K} k\epsilon(k,p_c^{\infty},n) \tag{S18}$$

The terms $\epsilon(k,p_c^{\infty},n)$ in the summation decay exponentially with n by rate $\alpha_k = |\ln[p_c^{\infty}/F(k)]|$, and because $n \to \infty$, the dominant term is the one with the lowest rate $\alpha = \min_k\{\alpha_k\}$, and the corresponding k will be denoted by k_{slow}, meaning $\alpha = \alpha_{k_{\text{slow}}}$.
Therefore,

$$p_c \sim p_c^{\infty} - A\frac{1}{n}e^{-\alpha n} \tag{S19}$$

where $\alpha = \min_k |\log[p_c^\infty/F(k)]|$ and $A = (2p_c^\infty k_{\rm slow})/(k_> k_<)$. It is obvious that $k_{\rm slow}$ is $k_<$ or $k_>$ because $F(k)$ is monotonic.

We know that if $F(k) = 1$ then $\epsilon = 0$, so if $F(k_{\rm slow}) = F(k_>) = 1$ then $k_<$ should be taken as $k_{\rm slow}$. It should also be noted that if $F(k_{\rm slow}) = p_c^\infty$ or very close, then $\epsilon(k_{\rm slow}, p_c^\infty, n) = -n^{-1}\log 2$. Another special case is where $k_{\rm slow}$ is not unique, then we take all of them, and $k_{\rm slow}$ is replaced by $\sum_{k_{\rm slow}} k_{\rm slow}$.

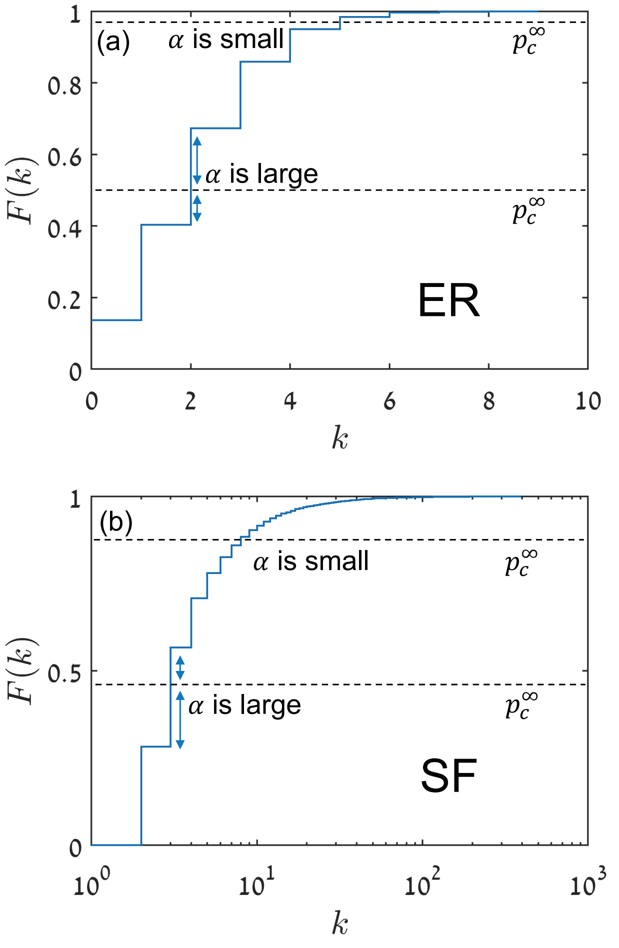

FIG. S2. **Examples of** $F(k)$**.** The decay rate α determines the regimes of power law or exponential convergence of $p_c(n \to \infty)$. α depends on the minimal ratio between p_c^∞ and $F(k)$. Hence α is influenced by the density of $F(k)$ in the neighborhood of p_c^∞. Here we show the patterns of $F(k)$ for ER network with $N = 10^4$ and $k = 2$ (a), and for SF network with $N = 10^4$ and $\gamma = 2.5$ (b). One can see that for larger p_c^∞, $F(k)$ is much more dense for SF network, which makes α become small and extend the power-law regime.

The behavior of Eq. (S19) depends on the value of n, and splits into two regimes. Where $n \ll 1/\alpha$, convergence will be according to a power-law, and where $n \gg 1/\alpha$ the convergence is exponential. It means that the width of these two regimes depends on α. The value of α depends on the value of p_c^∞ and the details of the degree distribution. Therefore, taking conditions providing $p_c^\infty \to 1$ might lead to a broader power law regime. That is because the tail of the degree distribution, $P(k)$, has very small values, which can be $\sim 1/N$. That implies that there are values of $F(k) \sim 1 - 1/N$, which yields $\alpha = \min_k |\log(p_c^\infty/F(k))| \approx \log(1) - \log(1-1/N) \approx 1/N$, and makes the power law regime being any $n \ll N$ in this case. In contrast, if p_c^∞ is not very close to 1, then α is expected to be finite because $F(k)$ in not very dense in this regime. Therefore, the exponential behavior will rule even for small n. See in Fig. S2 examples of ER and SF networks.

D. Derivation of p_c vs n for Scale-Free networks with small n

Here we consider SF network with $2 < \gamma < 3$. If $n = 1$, then $p_c \to 0$. In order to find out for which n, p_c becomes non-zero, we analyze Eqs. (S6) and (S16) for large k (high degrees govern the behavior in SF network), small n and p as follows.
It can be shown that

$$F(k) \approx 1 - (k/m)^{1-\gamma}\,, \tag{S20}$$

Then,

$$\begin{aligned} F(k)^n &\approx \left(1 - (k/m)^{1-\gamma}\right)^n \approx \exp\left[n\log\left(1 - (k/m)^{1-\gamma}\right)\right] \\ &\approx \exp\left[-n\,(k/m)^{1-\gamma}\right] \approx 1 - n\,(k/m)^{1-\gamma}\,, \\ F(k)^{1-n} &\approx 1 + (n-1)\,(k/m)^{1-\gamma}\,, \end{aligned}$$

where we assume $(k/m)^{\gamma-1} \gg n$ for large degrees. Plugging this into Eq. (S6),

$$\begin{aligned} F_p(k) &= \left(1 + \left(F(k)^{1-n} - 1\right)p^{n-1}\right)^{-\frac{1}{n-1}} \\ &\approx \left(1 + (n-1)\,(k/m)^{1-\gamma}\,p^{n-1}\right)^{-\frac{1}{n-1}} \\ &\approx \exp\left[-\frac{1}{n-1}\log\left(1 + (n-1)\,(k/m)^{1-\gamma}\,p^{n-1}\right)\right] \\ &\approx \exp\left[-\frac{1}{n-1}(n-1)\,(k/m)^{1-\gamma}\,p^{n-1}\right] \\ &\approx 1 - p^{n-1}\,(k/m)^{1-\gamma}\,. \end{aligned}$$

That leads to

$$P_p(k) \approx \frac{\partial F_p(k)}{\partial k} \approx \frac{\gamma-1}{m}p^{n-1}\left(\frac{k}{m}\right)^{-\gamma} = p^{n-1}P(k). \tag{S21}$$

Now we should notice that $P_p(k)$ has a new natural cutoff, K_p, which depends on p as following

$$\begin{aligned} \int_{K_p}^{\infty} p^{n-1}Ak^{-\gamma} &= \frac{1}{Np}, \\ K_p &\sim p^{n/(\gamma-1)}N^{1/(\gamma-1)}. \end{aligned} \tag{S22}$$

Taking the result of Eq. (S21) into Eq. (S16) and keeping the leading terms, where $\langle k_{p_c}\rangle$ (average degree of the remaining nodes) is finite and $\langle k_{p_c}^2\rangle$ increases with N, we obtain

$$1 = \frac{p_c}{\langle k\rangle}\sum_{k=0}^{\infty} k(k-1)P_{p_c}(k) \sim \frac{p_c}{\langle k\rangle}\langle k_{p_c}^2\rangle.$$

But

$$\langle k_{p_c}^2\rangle \sim \int_m^{K_{p_c}} k^2 p_c^{n-1} A k^{-\gamma} \sim p_c^{n-1} {K_{p_c}}^{3-\gamma} \sim p_c^{n-1+n\beta} N^\beta, \tag{S23}$$

where $\beta = (3-\gamma)/(\gamma-1)$.
Substituting $\langle k_{p_c}^2\rangle$ into the Eq. above provides

$$1 \sim \frac{p_c}{\langle k\rangle}\langle k_{p_c}^2\rangle \sim \frac{p_c^{n+n\beta}}{\langle k\rangle} N^\beta.$$

Therefore

$$p_c \sim N^{-\delta/n} \sim \exp\left[-\delta \log(N)/n\right], \tag{S24}$$

where

$$\delta = \frac{\beta}{1+\beta} = \frac{3-\gamma}{2}. \tag{S25}$$

This scaling is with N. The pre-factor depends on n but not in N. The scaling is valid for large N.
From Eq. (S24), it is easy to see that if $n \ll \log N$, $p_c \to 0$, while if $n \sim \log N$, p_c becomes non-zero.
After we completed the calculation, for consistency, we should return to check our initial assumption saying $(k/m)^{\gamma-1} \gg n$ for the large degrees. We should apply this condition on K_{p_c} that we found. $n \ll (K_{p_c}/m)^{\gamma-1} \sim p_c^n N/m^{\gamma-1}$, and substituting p_c that we found, we get $n \ll N^{1-\delta} = N^{(\gamma-1)/2}$. To conclude, we can say that Eq. (S24) is valid for

$$n \ll N^{(\gamma-1)/2}. \tag{S26}$$

For completeness, we will find now the pre-factor in Eq. (S24), $C(n)$. To this end, we go back to Eqs. (S22) and (S23) for getting

$$K_p \sim p^{n/(\gamma-1)}\left(\frac{AN}{\gamma-1}\right)^{1/(\gamma-1)}.$$

$$\langle k_{p_c}^2\rangle \sim \frac{A}{3-\gamma} p_c^{n-1+n\beta}\left(\frac{AN}{\gamma-1}\right)^{\beta},$$

Therefore,

$$1 \sim \frac{p_c}{\langle k\rangle}\langle k_{p_c}^2\rangle \sim \frac{A^{1+\beta}}{(3-\gamma)(\gamma-1)^\beta \langle k\rangle} p_c^{n+n\beta} N^\beta.$$

But,

$$A \approx (\gamma-1)m^{\gamma-1},$$

$$\langle k\rangle \approx \frac{A}{\gamma-2} m^{2-\gamma} \approx \frac{\gamma-1}{\gamma-2} m.$$

Thus,

$$1 \sim \left(\frac{\gamma-2}{3-\gamma} m\right) p_c^{n+n\beta} N^\beta.$$

Hence, we obtain now Eq. (S24) with the pre-factor

$$p_c \sim C(n) N^{-\delta/n},$$

where

$$C(n) = \phi^{1/n},$$

and

$$\phi = \left(\frac{3-\gamma}{\gamma-2}\frac{1}{m}\right)^{(\gamma-1)/2}.$$

III. EXAMPLE OF A SIMPLE NETWORK WITH ONLY TWO DEGREES

Here we consider an example of a simple degree distribution which can be analyzed simply as following

$$P(k) = \begin{cases} r, & k=1 \\ 1-r, & k=3 \\ 0, & \text{else} \end{cases} \tag{S27}$$

Then $F(1) = r,\ F(2) = r,\ F(3) = 1$.

Assuming that $r < p_c < 1$, one can observe that this assumption is consistent with the final result. Hence, from Eq. (S9)

$$F_p(k) = \begin{cases} 0, & k=0 \\ \frac{r}{p_c} - \frac{1}{n}\left(\frac{r}{p_c}\right)^{n+1}, & k=1 \\ \frac{r}{p_c} - \frac{1}{n}\left(\frac{r}{p_c}\right)^{n+1}, & k=2 \\ 1, & k=3 \end{cases} \tag{S28}$$

Thus

$$\frac{\langle k\rangle}{p_c} = \sum_{k=2}^{3} k(k-1)\Delta F_p(k) = 2\cdot 1\cdot 0 + 3\cdot 2\cdot\left[1 - \frac{r}{p_c} + \frac{1}{n}\left(\frac{r}{p_c}\right)^{n+1}\right],$$

$$\frac{3-2r}{p_c} = 6\left[1 - \frac{r}{p_c} + \frac{1}{n}\left(\frac{r}{p_c}\right)^{n+1}\right],$$

$$p_c = \frac{3+4r}{6} - \frac{r}{n}\left(\frac{r}{p_c}\right)^{n}.$$

For the limit of $n \to \infty$

$$p_c^\infty = \frac{1}{2} + \frac{2r}{3}. \tag{S29}$$

Note that $r < 3/4$, otherwise, there is no giant component even without attack.

Then, we conclude for large n that $p_c = p_c^\infty + o(1)$. As a result, for the leading term

$$p_c = p_c^\infty - \frac{r}{n}\left(\frac{r}{p_c^\infty}\right)^n = p_c^\infty - \frac{r}{n}\exp(-\alpha n), \tag{S30}$$

where $\alpha = \log(2/3 + 1/(2r))$.

The other limit of $n = 1$ is the random attack, and p_c is obtained by the simple known formula for configuration model $p_c = 1/(\kappa - 1)$. Is is easy to find that $\langle k \rangle = 3 - 2r$ and $\langle k^2 \rangle = 9 - 8r$, resulting

$$p_c(n = 1) = \frac{1}{2} + \frac{1}{6}\frac{r}{1-r}. \tag{S31}$$

Now we analyze a similar but a little bit different case where

$$P(k) = \begin{cases} r, & k = 2 \\ 1 - r, & k = 3 \\ 0, & \text{else} \end{cases} \tag{S32}$$

Here $F(1) = 0,\ F(2) = r,\ F(3) = 1$. Thus,

$$F_p(k) = \begin{cases} 0, & k = 1 \\ \frac{r}{p_c} - \frac{1}{n}\left(\frac{r}{p_c}\right)^{n+1}, & k = 2 \\ 1, & k = 3 \end{cases} \tag{S33}$$

Therefore,

$$\frac{\langle k \rangle}{p_c} = \sum_{k=2}^{3} k(k-1)\Delta F_p(k) = 2 \cdot 1 \cdot \left[\frac{r}{p_c} - \frac{1}{n}\left(\frac{r}{p_c}\right)^{n+1}\right] + 3 \cdot 2 \cdot \left[1 - \frac{r}{p_c} + \frac{1}{n}\left(\frac{r}{p_c}\right)^{n+1}\right],$$

$$\frac{3-r}{p_c} = 6 - 4\left[\frac{r}{p_c} - \frac{1}{n}\left(\frac{r}{p_c}\right)^{n+1}\right],$$

$$p_c = \frac{1+r}{2} - \frac{2r}{3n}\left(\frac{r}{p_c}\right)^n.$$

For the limit of $n \to \infty$

$$p_c^\infty = \frac{1}{2} + \frac{r}{2}. \tag{S34}$$

Therefore, for the next leading term

$$p_c = \frac{1+r}{2} - \frac{2r}{3n}\left(\frac{2r}{1+r}\right)^n. \tag{S35}$$

Now, if we will take $r \sim 1 - b/N$, where b is finite, then

$$p_c \approx 1 - \frac{2}{3n}\left(1 - \frac{b}{N}\right)^n \approx 1 - \frac{2}{3n}e^{-b\frac{n}{N}}. \tag{S36}$$

Hence, if $n \ll N$ then

$$1 - p_c \sim \frac{1}{n}. \tag{S37}$$

This happens because $p_c \to 1$ where $r \to 1$, but $F(2) = r$, hence $F(2) \to p_c$, which yields a power law behavior of $p_c(n)$.

TABLE II. Structure features of six real-world networks.

Name	N	E	$< k >$
Skitter	1696415	11095298	13.08
ia-digg-reply	29652	84781	6.40
as-CAIDA	26475	53381	4.03
Douban	154908	327162	4.22
tech-pep	62561	147878	4.73
sc9	5921786	23667183	7.99

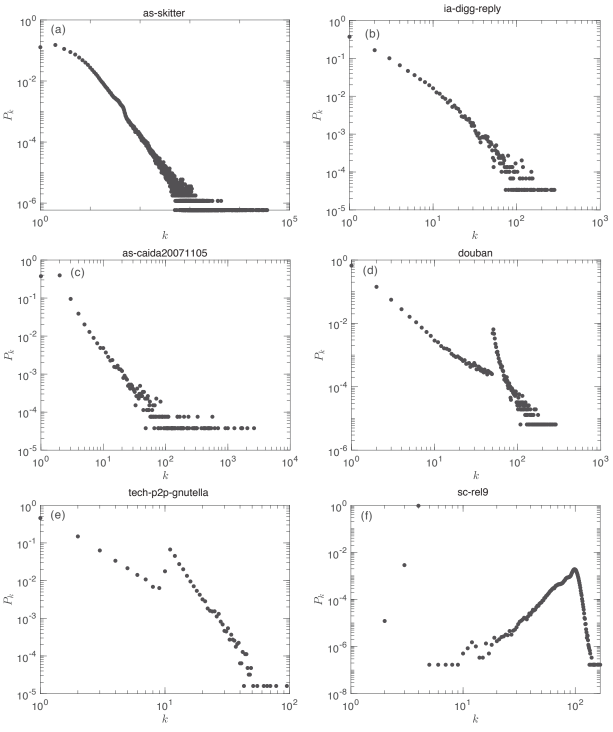

FIG. S3. The degree distribution of real networks. The first three networks show scale-free characters while the last three are not.

IV. REAL DATA

We tested our approach on the six real-world networks from different field ranging from computers, collaboration and large on-line social networks as shown fig. S3.

Particularly, they are (a) Skitter network, the undirected network of autonomous system on the Internet connected to each other, from the Skitter project [1]. (b) ia-digg-reply. This is a reply network of the social news website Digg. Nodes are users of the website and each directed edge denotes that a user replied to another user [2, 3]. (c) as-CAIDA, the undirected network of autonomous systems of the Internet connected with each other from the CAIDA project, collected in 2007. Nodes are autonomous systems (AS), and edges denote communication [1]. (d) Douban, the social fridenship network of douban, a Chinese online recommendation site. The network is undirected and unweighted [4]. (e) tech-p2p, eDonkey peer to peer network [3, 5]. (f) sc-rel9, scientific computing network [3, 6]. From fig. S3, one can observe that these three networks following scale-free features while for other three do not. The structure features are summarized in Table. II. N and E are the number of nodes and edges in the network respectively. $<k>$ is the average degree of networks.

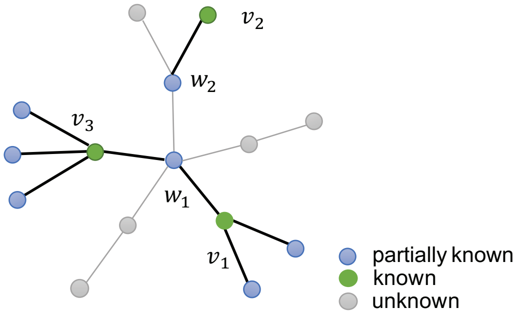

FIG. S4. Illustration of model-II. Assuming $n = 3$, and in a specific step, nodes v_1, v_2, v_3 are known and their associated neighbors (marked in blue) are partially known, $d_{w_1} = 2, d_{w_2} = 1$. The highest degree node among the known nodes and the partially known nodes are chosen to be immunized.

V. DISCUSSION OF A SECOND MODEL

In our model, we assume that the preventer obtains each time the degree information of n nodes. However, in most cases, if we know a node's degree, we can know partial degree information of its neighbor nodes. For example, in Fig. S4, three nodes v_1, v_2, v_3 are the known nodes. Accordingly, nodes marked in blue are partially known for us, such as the degree of w_1 and w_2 are at least 2 and 1 respectively. Therefore, the highest degree node here can be chosen from the known and the partially known nodes. In the following, we refer to this model as model-II while the model in the maintext as model-I. We find that, actually, adding partially information about neighboring nodes has almost no affect on the final results.

As seen in Fig. S4, the chosen node in both model-I

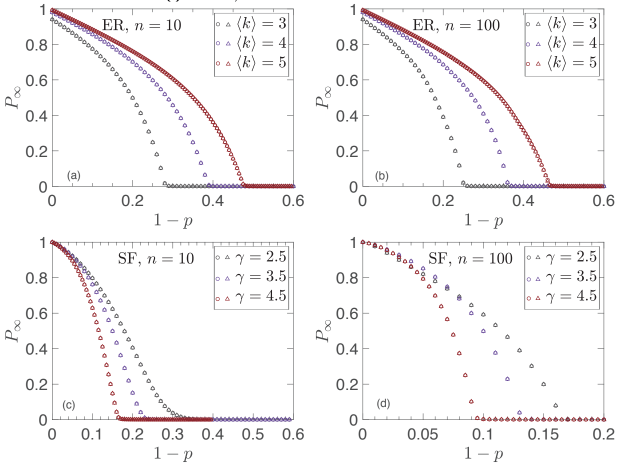

FIG. S5. The comparison of model-I and model-II for ER (a-b) and SF (c-d) networks. Circles are the results of model-I while triangles are model-II. In panels (a)-(b), from left to right, they are $\langle k \rangle = 3, 4, 5$ for different n respectively. In panels (c)-(d), from left to right, they are $\gamma = 4.5, 3.5, 2.5$ for different n respectively. The network size is 10^6 and simulation results are averaged over 100 independent realizations.

and model-II is the same node v_1. The degree of most partially known neighbors is very low and usually 1. Indeed, the likelihood of the case where more than one chosen node share a common neighbor like node w_1 decrease as the network size increase. In Fig. S5, we compare the giant component as a function of $1-p$ for both models (I and II) for ER and SF networks respectively. Our results demonstrates that the final results are nearly the same.

[1] Jure Leskovec, Jon Kleinberg, and Christos Faloutsos. Graph evolution: Densification and shrinking diameters. *ACM Transactions on Knowledge Discovery from Data (TKDD)*, 1(1):2, 2007.

[2] Munmun De Choudhury, Hari Sundaram, Ajita John, and Dorée Duncan Seligmann. Social synchrony: Predicting mimicry of user actions in online social media. In *International Conference on Computational Science and Engineering*, volume 4, pages 151–158. IEEE, 2009.

[3] Ryan A. Rossi and Nesreen K. Ahmed. The network data

repository with interactive graph analytics and visualization. In *AAAI*, 2015.
[4] Reza Zafarani and Huan Liu. Social computing data repository at asu, 2009.
[5] Tore Opsahl and Pietro Panzarasa. Clustering in weighted networks. *Social networks*, 31(2):155–163, 2009.
[6] David A Bader, Henning Meyerhenke, Peter Sanders, and Dorothea Wagner. Graph partitioning and graph clustering. In *10th DIMACS Implementation Challenge Workshop*, 2012.
